# KAT6A regulates stemness of aging bone marrow-derived mesenchymal stem cells through Nrf2/ARE signaling pathway

**DOI:** 10.1186/s13287-021-02164-5

**Published:** 2021-02-04

**Authors:** Dongdong Fei, Yazheng Wang, Qiming Zhai, Xige Zhang, Yang Zhang, Yang Wang, Bei Li, Qintao Wang

**Affiliations:** 1 State Key Laboratory of Military Stomatology & National Clinical Research Center for Oral Diseases & Shaanxi Engineering Research Center for Dental Materials and Advanced Manufacture, Department of Periodontology, School of Stomatology, The Fourth Military Medical University, Xi’an, 710032 Shaanxi China; 2 State Key Laboratory of Military Stomatology & National Clinical Research Center for Oral Diseases & Shaanxi International Joint Research Center for Oral Diseases, Center for Tissue Engineering, School of Stomatology, The Fourth Military Medical University, Xi’an, 710032 Shaanxi China

**Keywords:** KAT6A, Nrf2/ARE signaling pathway, Aging, Bone marrow-derived mesenchymal stem cells, Stemness

## Abstract

**Background:**

This study aimed to explore the effect of KAT6A on the decreased stemness of aging bone marrow-derived mesenchymal stem cells (BMSCs) and its potential mechanism.

**Methods:**

The acetylation level and KAT6A expression of BMSCs from the young (YBMSCs) and the old (OBMSCs) were examined. Gain- and loss-of-function experiments were performed to determine the effect of KAT6A on BMSC proliferation, colony formation, and osteogenic differentiation. The effect of KAT6A on Nrf2/ARE signaling pathway was investigated after KAT6A inhibition in YBMSCs or overexpression in OBMSCs, and the role of Nrf2/ARE signaling pathway on stemness was examined by investigating proliferation, colony formation, and osteogenic differentiation. Further in vivo study was performed to explore osteogenesis ability of OBMSCs after modulation of KAT6A and Nrf2/ARE pathway through cell sheet technology.

**Results:**

The acetylation level and KAT6A expression of OBMSCs were decreased, and KAT6A downregulation resulted in decreased proliferation, colony formation, and osteogenic differentiation of OBMSCs. Mechanically, KAT6A was found to regulate Nrf2/ARE signaling pathway and inhibit ROS accumulation in OBMSCs, thus promoting proliferation, colony formation, and osteogenic differentiation of OBMSCs. Further study demonstrated that KAT6A could promote osteogenesis of OBMSCs by regulating Nrf2/ARE signaling pathway.

**Conclusions:**

Downregulation of KAT6A resulted in the decreased stemness of OBMSCs by inhibiting the Nrf2/ARE signaling pathway.

**Graphical abstract:**

KAT6A was downregulated in aging bone marrow-derived mesenchymal stem cells (BMSCs), and downregulation of KAT6A resulted in Nrf2/ARE signaling pathway inhibition and ROS accumulation, thus leading to decreased stemness of aging BMSCs. 
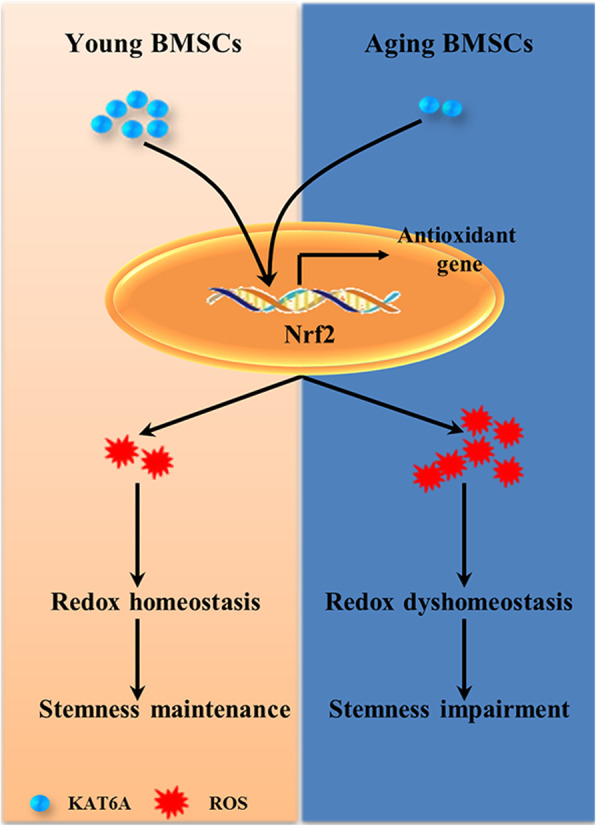

**Supplementary Information:**

The online version contains supplementary material available at 10.1186/s13287-021-02164-5.

## Background

Age-related osteoporosis, characterized by bone fragility, has become one of the main risks that endanger the health of the elderly. Bone marrow-derived mesenchymal stem cells (BMSCs) are essential for bone formation, and decreased stemness of BMSCs has been regarded as one important factor contributing to age-related osteoporosis [1, 2]. Numerous studies have shed light on the mechanism of decreased stemness of BMSCs during aging; however, how aging leads to this still needs further investigation.

Epigenetic alteration, including changes of methylation and acetylation of DNA and histones, has been regarded as one hallmark of aging through changing genomic instability and gene expression profiles [3, 4]. Histone acetyltransferases (HATs), such as KAT2A, elongator complex protein 3, and KAT6A, are enzymes responsible for histone lysine residues acetylation. They have numerous functions including DNA repair and transcriptional regulation [5]. Several studies have also shown that HATs can regulate stemness of MSCs [6–8]. The KAT6A gene, which can acetylate lysine residues in histone H3, is required for hematopoiesis [9] and skeletogenesis, as well as the regulation of many genes. Previous study has indicated that KAT6A could act as a transcriptional coactivator for RUNX family transcription factor 2 (Runx2), and dysregulation of KAT6A can lead to impaired skeletogenesis. Besides, KAT6A was also found to regulate cellular senescence [10–12]. Several studies reported that KAT6A could regulate the self-renewal, proliferation, and differentiation of hematopoietic stem cells [9]; however, whether and how KAT6A regulated the decreased stemness of aging BMSCs remains unknown.

Oxidative stress, a situation that occurs when the accumulation of reactive oxygen species (ROS) overwhelms the antioxidant capacity, is related to the development of several diseases and has become an important predisposing factor for stem cell aging and age-related diseases [13–15]. When confronted with oxidative stress, transcription factor nuclear factor erythroid 2-related factor 2 (Nrf2) can accumulate in the nucleus and bind to the antioxidant response element to promote the expressions of antioxidant enzymes to maintain a stable internal environment [16]. Studies confirmed that decreased Nrf2/ARE signaling pathway can lead to stem cell dysfunction [17, 18], but the detailed mechanism needs further investigation. Some studies have shown that HATs can regulate Nrf2/ARE signaling pathway [19, 20], and one study reported that MOF, a HAT belonging to the MYST family, could promote nuclear retention of Nrf2 [21]. Similar to MOF, KAT6A also belongs to the MYST family and shares some structural similarities with MOF, indicating that KAT6A may also be implicated in the regulation of Nrf2/ARE signaling pathway. In our study, decreased KAT6A expression in OBMSCs was found, and downregulated KAT6A expression was demonstrated to contribute to the decreased stemness of OBMSCs. Mechanically, KAT6A was found to scavenge accumulated ROS in OBMSCs through the Nrf2/ARE signaling pathway, thus ameliorating stemness and improving osteogenesis of OBMSCs.

## Methods

### Cell culture

Human BMSCs collected from the First Hospital Affiliated to the Fourth Military Medical University were used in this study with the approval of the Ethics Committee of the Fourth Military Medical University. YBMSCs were from persons who are under 30 years old, and OBMSCs were from those who are over 55 years old. To isolate human BMSCs, bone marrow samples from the iliac were diluted using Dulbecco’s phosphate-buffered saline, followed by centrifugation for 30 min at a speed of 2000 rpm. Then, the cells were plated in the 10-cm dish and were cultured in the α-MEM supplemented with 10% fetal bovine serum (FBS; Thermo Electron, Melbourne, Australia), 100 U/ml penicillin, 100 mg/ml streptomycin, and 0.292 mg/ml glutamine (Invitrogen, Carlsbad, CA, USA). BMSCs at passages 3–6 were used, and the medium was refreshed every 3 days. ML 385 (R & D Systems, 6243, MI, USA), a specific Nrf2 inhibitor, was dissolved in DMSO. For the culture of BMSC sheets, 1 × 10^6^ cells were seeded on six-well dishes and cell sheet-induction medium (50 μg/ml vitamin C) was added to promote cell sheet formation after being cultured for 24 h. After 14 days, BMSC sheets were collected for animal study.

### Colony-forming unit-fibroblastic (CFU-F) assay

BMSCs treated at a density of 2 × 10^3^ were seeded in a 5-cm dish, and the medium was refreshed every 3 days. After 12 days of culture, the dish was washed using PBS twice and was fixed with 4% paraformaldehyde (Sigma-Aldrich Corp, St Louis, MO, USA) for 30 min. Then, 2% crystal violet (Amresco, Solon, OH, USA) was used to stain the clones. Clone that contains more than 50 cells under the stereomicroscope was defined as a colony.

### Cell proliferation assay

BMSCs were seeded in 96-well plates at a density of 2 × 10 [3]. After 12–24 h, 10 μl enhanced Cell Counting Kit-8 (CCK-8) (Beyotime, China) was added to each well and cells were transfected with corresponding reagents. The absorbance values at day 1, day 3, day 5, and day 7 were measured at 450 nm using a microplate reader.

### Osteogenic differentiation assay

BMSCs treated at a density of 2 × 10^5^ were plated on 12-well dishes and were cultured in the α-MEM until 80~90% confluence. BMSCs were then cultured under an osteogenic culture medium supplemented with 100 μg/ml ascorbic acid (Sigma, USA), 10 nmol/l dexamethasone (Sigma, USA), and 5 mmol/l β-glycerophosphate (Sigma, USA). After osteogenic induction for 14 days, quantitative reverse transcription polymerase chain reaction (qRT-PCR) was performed to explore the expressions of *runt-related transcription factor 2* (*Runx2*), *osteocalcin* (*OCN*), and *bone morphogenetic protein 2* (*BMP2*). After osteogenic induction for 21 days, calcium deposit was assessed using alizarin red staining, and 10% cetylpyridinium chloride was used for quantitative analysis at 562-nm absorbance value.

### Transfection assay

BMSCs treated at a density of 2 × 10^5^ were plated on 12-well dishes until 80~90% confluence followed by serum starvation for 2 h. For siRNA experiments, BMSCs at the experiment groups were transfected with a final concentration of 50 nM KAT6A siRNA (Ribobio, Guangzhou, China) or Nrf2 siRNA (Santa Cruz Biotechnology, sc-37030, Texas, USA), with scrambled siRNA (Santa Cruz Biotechnology, sc-37007, USA) at the control groups. For overexpression experiments, BMSCs at the experiment groups were transfected with 500 ng plasmid of KAT6A (OBiO Technology, Shanghai, China), with control overexpression vectors at the control groups. The transfection reagent Lipofectamine 2000 (Invitrogen, USA) was used as per the manufacturer’s instructions. After transfection, the culture medium was replaced by growth culture medium and BMSCs were harvested at 48 h for RNA and 72 h for protein detection. For osteogenic differentiation assay, the osteogenic induction medium was used in the following day.

### Quantitative RT-PCR

TRIzol Reagent TM (Invitrogen, USA) was used to extract total cellular RNA as per the manufacturer’s instructions, and cDNA was synthesized in a 20-μl reaction volume. The qRT-PCR reactions were performed using the SYBR Premix Ex TaqTMII kit (Takara, Tokyo, Japan), and CFX96 Touch Real-Time System (Bio-Rad, CA, USA) was used for detection. Fold changes of mRNA were calculated using the 2^−△△Ct^ method with the reference gene glyceraldehyde-3-phosphate dehydrogenase (GAPDH). The primer sequences are listed in Supplementary Table S1 in Additional file 1.

### Western blot analysis

Cells were lysed in RIPA lysis buffer (Beyotime Institute of Biotechnology, Shanghai, China) after being washed with PBS twice. To prepare the cytoplasmic component and nuclear component, BMSCs were lysed using nuclear and cytoplasmic protein extraction kit (Beyotime Institute of Biotechnology, China) as per manufacturer’s instructions. After separation and transferring using 10% Tris-glycine SDS-polyacrylamide gels and PVDF membranes, samples were blocked using 5% bovine serum albumin for 2 h. Then, PVDF membranes were incubated overnight at 4 °C with the following primary antibodies: anti-GAPDH (CWBIO, CW0100, Beijing, China), anti-β-tubulin (CWBIO, CW0098, China), anti-KAT6A (Santa Cruz Biotechnology, sc-5713, USA), anti-acetylated-lysine (Cell Signaling Technology, #9441, Beverly, MA, USA), anti-Histone H3 (Cell Signaling Technology, #9715, USA), anti-acetyl-Histone H3 (Millipore, 17-615, Billerica, CA, USA), anti-Histone H4 (Abcam, ab177840, Cambridge, UK), anti-acetyl-Histone H4 (R & D Systems, AF5215-SP, USA), anti-Histone H3 (acetyl K9) (Abcam, ab10812, UK), anti-Histone H3 (acetyl K14) (Abcam, ab52946, UK), and anti-Nrf2 (Cell Signaling Technology, #12721, USA). After incubation with secondary antibody (Jackson, West Grove, PA, USA) for 2 h at 37 °C, membranes were visualized using the enhanced chemiluminescence kit (Pierce, IL, USA). ImageJ (Media Cybernetics, MD, USA) was used for quantitative analysis.

### ROS detection

BMSCs were seeded in 96-well plates at a density of 2 × 10^4^ and were cultured using α-MEM supplemented with 10% FBS and 0.292 mg/ml glutamine. After 12–24 h, cells were transfected with corresponding reagents. In detail, for siRNA experiments, BMSCs were transfected with KAT6A siRNA, Nrf2 siRNA, or scrambled siRNA with a final concentration of 50 nM. For overexpression experiments, BMSCs were transfected with 50 ng plasmid of KAT6A or control overexpression vectors. For studies involving transfection of siRNA and plasmid, 50 nM siRNA and 50 ng plasmid were mixed and transfected at the same time. After transfection for 24 h, the medium was changed using 100 μl ROS red working solution (Abcam, ab186027, UK). After incubation at 37 °C for 60 min, a fluorescence microplate reader (PerkinElmer, MA, USA) was used to detect the intensity of fluorescence at 520-nm and 605-nm excitation and emission wavelengths, respectively.

### Animal study

The animal study was conducted according to the committee guidelines of the Animal Care Committee of the Fourth Military Medical University. Eight-week-old Sprague-Dawley (SD) male rats were randomly assigned to three groups with each group containing seven or eight rats. The surgical procedure of the alveolar bone defects was described as previously mentioned [22]. Briefly, SD rats were anesthetized by intraperitoneal administration of 1% pentobarbital sodium solution. After being shaved by a razor and having disinfected with iodophor, the entire skin along the lower edge of the unilateral mandible was cut. Then, the masseter muscle was cut off to expose the buccal side of the mandible, and a dental handpiece was used to remove the alveolar bone between the first and second molars about 1.0 to 1.5 mm away from the top of the alveolar ridge to make a periodontal defect with 3 mm long, 2 mm wide, and 1 mm thickness. BMSC sheets (OBMSCs + DMSO + vector, OBMSCs + DMSO + KAT6A plasmid, and OBMSCs + 10 μm ML 385 + KAT6A plasmid) were then filled into the defects. After 6 weeks, the SD rats were euthanized and the samples were collected for micro-CT analysis.

### Statistical analyses

All data are displayed as mean ± SD. Comparisons between the two groups were performed using two-tailed Student’s *t* test, and comparisons among the three groups were performed using one-way ANOVA. SPSS13.0 or GraphPad 5 was used, and significance was confirmed at *P* < 0.05 (*P < 0.05; **P < 0.01; ***P < 0.001).

## Results

### The KAT6A expressions were decreased in OBMSCs

Firstly, the stemness of YBMSCs and OBMSCs was investigated. CCK-8 and crystal violet analysis found lower proliferative and self-renewal capacity of OBMSCs than that of YBMSCs (Figure S1A and S1B). Besides, qRT-PCR and alizarin red staining confirmed the decreased osteogenesis ability of OBMSCs than that of YBMSCs (Figure S1C and S1D). These results indicated that OBMSCs exhibited lower stemness than YBMSCs. For more details, please refer to Additional file 2.

**Fig. 1:**
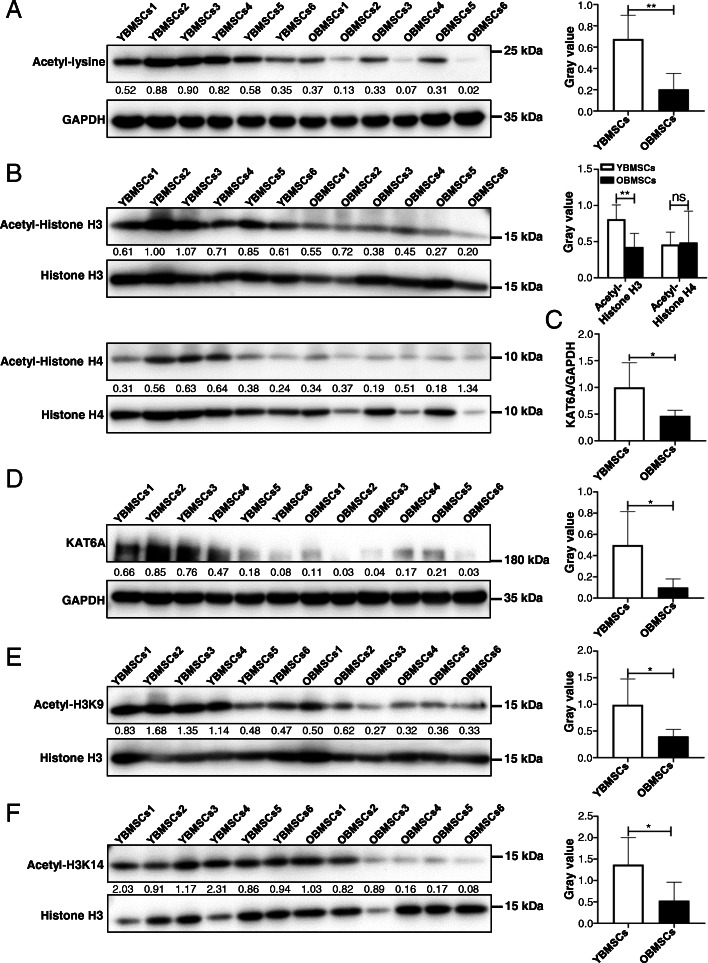
The KAT6A expressions were decreased in aging BMSCs. **a** The acetylation levels of lysine in YBMSCs and OBMSCs were explored by western blot, and quantitative analysis was performed by ImageJ (*n* = 6). **b** The expressions of Histone H3, acetyl-Histone H3, Histone H4, and acetyl-Histone H4 were explored by western blot, and quantitative analysis was performed by ImageJ (*n* = 6). **c** The expressions of KAT6A mRNA in YBMSCs and OBMSCs were explored by qRT-PCR (*n* = 6). **d** The expressions of KAT6A protein in YBMSCs and OBMSCs were explored by western blot, and quantitative analysis was performed by ImageJ (*n* = 6). **e** The acetylation levels of H3K9 in YBMSCs and OBMSCs were explored by western blot, and quantitative analysis was performed by ImageJ (*n* = 6). **f** The acetylation levels of H3K14 in YBMSCs and OBMSCs were explored by western blot, and quantitative analysis was performed by ImageJ (*n* = 6)

Then, the levels of acetylated lysine in YBMSCs and OBMSCs were explored, and the acetylation levels of lysine were found to be decreased in OBMSCs compared to those in YBMSCs (Fig. 1a), indicating that histone acetylation may be involved in the decreased stemness of OBMSCs. Moreover, histone H3 and histone H4 acetylation levels were examined, and western blot analysis showed decreased histone H3 acetylation levels in OBMSCs, while no significant difference was found in histone H4 acetylation levels (Fig. 1b). KAT6A is a HAT that is specific for acetylation of histone H3. The mRNA expressions of KAT6A in YBMSCs and OBMSCs were investigated, and qRT-PCR results showed that KAT6A mRNA expressions were downregulated in OBMSCs compared to that in YBMSCs (Fig. 1c). Downregulated KAT6A expressions in OBMSCs were also confirmed by Western blot (Fig. 1d). To further confirm the effect of decreased KAT6A expressions on OBMSCs, KAT6A-specific acetylation sites H3K9 [10] and H3K14 [23] were examined, and decreased acetyl-H3K9 and acetyl-H3K14 in OBMSCs were found (Fig. 1e, f). All these results proved that KAT6A expressions were decreased in OBMSCs.

### KAT6A regulated stemness of BMSCs

**Fig. 2:**
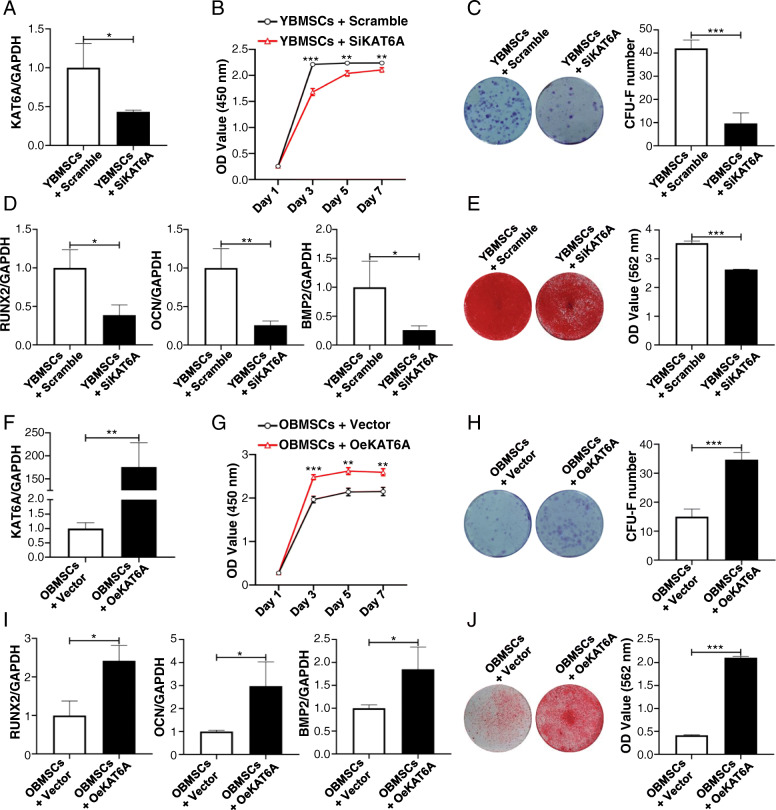
KAT6A regulated stemness of OBMSCs. **a** Forty-eight hours later, the transfection efficiency was tested by qRT-PCR after transfection of scrambled siRNA or KAT6A siRNA into YBMSCs (*n = 3*). **b** CCK-8 was performed to explore the proliferative capacity of YBMSCs that transfected with scrambled siRNA or KAT6A siRNA (*n = 3*). **c** The colony-forming abilities of YBMSCs that transfected with scrambled siRNA or KAT6A siRNA were explored by crystal violet after culture for 12 days (*n = 3*). **d** After osteogenic induction for 14 days, expressions of osteogenic-related genes of *Runx2*, *OCN*, and *BMP2* were detected in YBMSCs that transfected with scrambled siRNA or KAT6A siRNA (*n = 3*). **e** After osteogenic induction for 21 days, alizarin red staining was performed in YBMSCs that transfected with scrambled siRNA or KAT6A siRNA (*n = 3*). **f** Forty-eight hours later, the transfection efficiency was tested by qRT-PCR after transfection of vector or KAT6A plasmid into OBMSCs (*n* = 3). **g** CCK-8 was performed to explore the proliferative capacity of OBMSCs that transfected with vector or KAT6A plasmid (*n = 3*). **h** The colony-forming abilities of OBMSCs that transfected with vector or KAT6A plasmid were explored by crystal violet after culture for 12 days (*n = 3*). **i** After osteogenic induction for 14 days, expressions of osteogenic-related genes of *Runx2*, *OCN*, and *BMP2* were detected in OBMSCs that transfected with vector or KAT6A plasmid (*n* = 3). **j** After osteogenic induction for 21 days, alizarin red staining was performed in OBMSCs that transfected with vector or KAT6A plasmid (*n = 3*)

To demonstrate whether KAT6A regulated stemness of BMSCs, KAT6A expression in YBMSCs was downregulated, and KAT6A expression in OBMSCs was upregulated through KAT6A siRNA and overexpression plasmid. Transfection efficiencies of inhibition and overexpression were confirmed using qRT-PCR (Fig. 2a, f). After KAT6A inhibition, the proliferative capacity of KAT6A-inhibited YBMSCs was lower than that of the scramble-treated YBMSCs (Fig. 2b). Additionally, the colony-forming ability of YBMSCs was also decreased after KAT6A inhibition (Fig. 2c). Besides, osteogenic-related genes of *Runx2*, *OCN*, and *BMP2* were found to be downregulated after KAT6A inhibition in YBMSCs as confirmed by qRT-PCR (Fig. 2d). Alizarin red staining further confirmed decreased mineral node formation in KAT6A-inhibited YBMSCs (Fig. 2e). Conversely, KAT6A overexpression could improve the proliferative capacity and the colony-forming ability of OBMSCs (Fig. 2g, h). Besides, KAT6A overexpression could increase the expressions of osteogenic-related genes of *Runx2*, *OCN*, and *BMP2* (Fig. 2i) and promote more mineral node formation of OBMSCs (Fig. 2j).

### Decreased KAT6A expression led to ROS accumulation in OBMSCs by inhibiting Nrf2/ARE signaling pathway

**Fig. 3:**
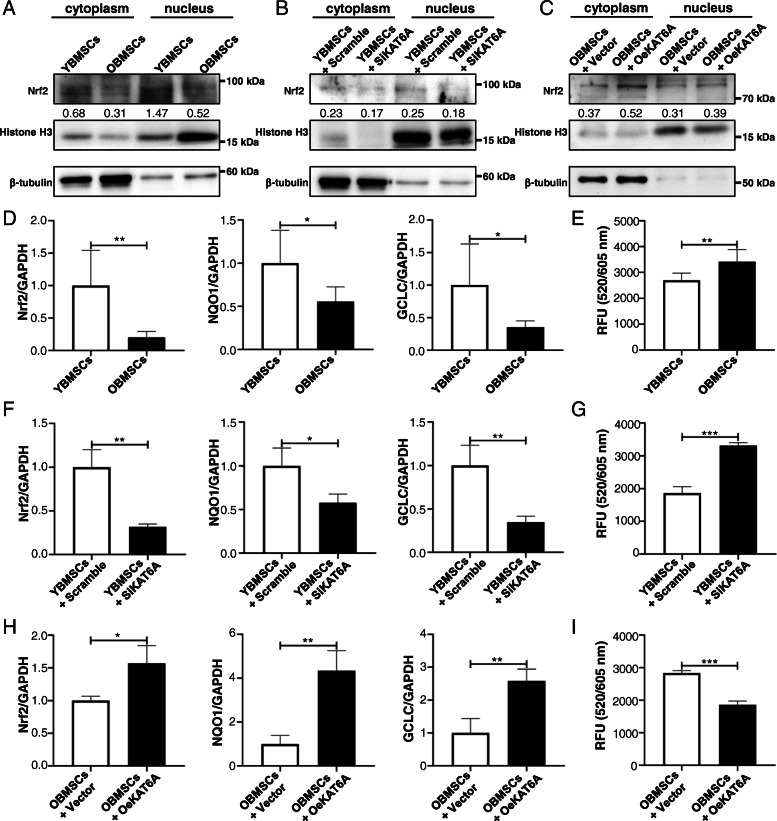
Decreased KAT6A expression inhibited Nrf2/ARE signaling pathway and led to ROS accumulation in OBMSCs. **a** The expressions of Nrf2 proteins in YBMSCs and OBMSCs were explored by western blot (*n* = 6). **b** The expressions of Nrf2 protein in YBMSCs that transfected with scrambled siRNA or KAT6A siRNA were explored by western blot (*n* = 3). **c** The expressions of Nrf2 protein in OBMSCs that transfected with vector or KAT6A plasmid were explored by western blot (*n* = 3). **d** The expressions of *Nrf2*, *GCLC*, and *NQO1* in YBMSCs and OBMSCs were explored by qRT-PCR (*n* = 6). **e** The ROS level in YBMSCs and OBMSCs was explored (*n* = 6). **f** The expressions of *Nrf2*, *GCLC*, and *NQO1* in YBMSCs that transfected with scrambled siRNA or KAT6A siRNA were explored by qRT-PCR (*n* = 3). **g** The ROS level in YBMSCs that transfected with scrambled siRNA or KAT6A siRNA was explored (*n* = 3). **h** The expressions of *Nrf2*, *GCLC*, and *NQO1* in OBMSCs that transfected with vector or KAT6A plasmid were explored by qRT-PCR (*n* = 3). **i** The ROS level in OBMSCs that transfected with vector or KAT6A plasmid was explored (*n* = 3)

The accumulation of ROS during the aging process can contribute to stem cell dysfunction, and Nrf2/ARE signaling pathway is responsible for ROS scavenging. Several studies demonstrated that HATs could regulate Nrf2/ARE signaling pathway. Thus, KAT6A was suspected to improve stemness of BMSCs through Nrf2/ARE signaling pathway. In this study, accumulated ROS were found in OBMSCs (Fig. 3e), and intranuclear Nrf2 level in OBMSCs was downregulated as confirmed by western blot (Fig. 3a). Moreover, the target genes of Nrf2/ARE signaling pathway, *glutamate-cysteine ligase catalytic subunit* (*GCLC*) and *NAD(P)H quinone dehydrogenase 1* (*NQO1*), were also downregulated in OBMSCs (Fig. 3d). All these results indicated that Nrf2/ARE signaling pathway was inhibited during the aging process. Then, KAT6A was inhibited in YBMSCs and KAT6A was overexpressed in OBMSCs, and the results showed that decreased KAT6A expression in OBMSCs could result in ROS accumulation, as KAT6A-inhibited YBMSCs presented more ROS accumulation and KAT6A-overexpressed OBMSCs presented less ROS accumulation (Fig. 3g, i). Meanwhile, less intranuclear Nrf2 expressions were detected after KAT6A inhibition in YBMSCs (Fig. 3b), while more intranuclear Nrf2 expressions were detected after KAT6A overexpression in OBMSCs (Fig. 3c). Besides, the target genes of Nrf2/ARE signaling pathway were also downregulated in KAT6A-inhibited YBMSCs and were upregulated in KAT6A-overexpressed OBMSCs (Fig. 3f, h). These results supported that KAT6A could regulate Nrf2/ARE signaling pathway.

### KAT6A regulated stemness of OBMSCs through Nrf2/ARE signaling pathway

**Fig. 4:**
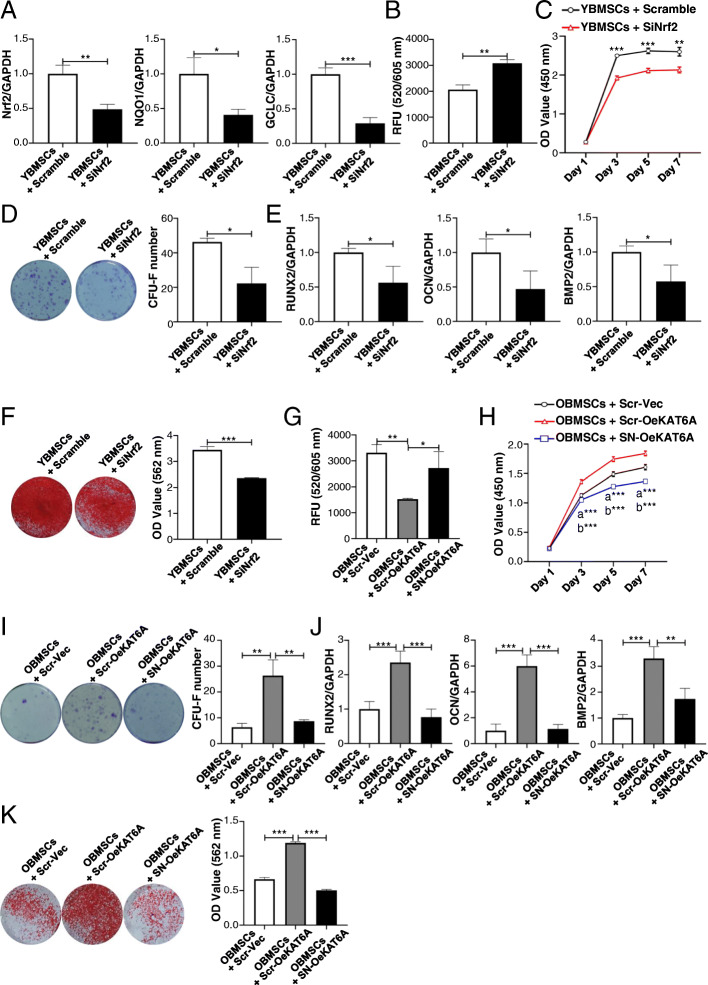
KAT6A regulated stemness of OBMSCs through Nrf2/ARE signaling pathway. **a** Forty-eight hours later, the expressions of *Nrf2*, *GCLC*, and *NQO1* in YBMSCs that transfected with scrambled siRNA or Nrf2 siRNA were explored by qRT-PCR (*n* = 3). **b** The ROS level in YBMSCs that transfected with scrambled siRNA or Nrf2 siRNA was explored (*n* = 3). **c** CCK-8 was performed to explore the proliferative capacity of YBMSCs that transfected with scrambled siRNA or Nrf2 siRNA (*n* = 3). **d** The colony-forming abilities of YBMSCs that transfected with scrambled siRNA or Nrf2 siRNA were explored by crystal violet after culture for 12 days (*n* = 3). **e** After osteogenic induction for 14 days, expressions of osteogenic-related genes of *Runx2*, *OCN*, and *BMP2* were detected in YBMSCs that transfected with scrambled siRNA or Nrf2 siRNA (*n* = 3). **f** After osteogenic induction for 21 days, alizarin red staining was performed in YBMSCs that transfected with scrambled siRNA or Nrf2 siRNA (*n* = 3). **g** After overexpressing KAT6A in OBMSCs in the content of Nrf2 siRNA, the ROS level was explored (*n* = 3). **h** After overexpressing KAT6A in OBMSCs in the content of Nrf2 siRNA, CCK-8 was performed to explore the proliferative capacity (*n* = 3). **i** After overexpressing KAT6A in OBMSCs in the content of Nrf2 siRNA, the colony-forming abilities were explored by crystal violet after culture for 12 days (*n* = 3). **j** After overexpressing KAT6A in OBMSCs in the content of Nrf2 siRNA, expressions of osteogenic-related genes of *Runx2*, *OCN*, and *BMP2* were detected (*n* = 3). **k** After overexpressing KAT6A in OBMSCs in the content of Nrf2 siRNA, alizarin red staining was performed (*n* = 3). Scr-Vec, scramble + vector; Scr-OeKAT6A, scramble + OeKAT6A; SN-OeKAT6A, SiNrf2 + OeKAT6A; **a** “OBMSCs + Scr-Vec” compared with “OBMSCs + Scr-OeKAT6A”; **b** “OBMSCs + Scr-OeKAT6A” compared with “OBMSCs + SN-OeKAT6A”

To demonstrate that KAT6A regulated stemness of OBMSCs through Nrf2/ARE signaling pathway, first, Nrf2/ARE signaling pathway was inhibited to explore the relationship between the Nrf2/ARE signaling pathway and the decreased stemness of OBMSCs. Nrf2 siRNA was used to inhibit Nrf2/ARE signaling pathway of YBMSCs (Fig. 4a), and the results showed that the inhibition of Nrf2/ARE signaling pathway could increase ROS level in YBMSCs (Fig. 4b). After Nrf2 inhibition, the proliferative capacity of YBMSCs was decreased (Fig. 4c), and the colony-forming ability was also downregulated (Fig. 4d). Besides, results from qRT-PCR and alizarin red staining showed that the osteogenic differentiation of YBMSCs was inhibited after Nrf2 inhibition (Fig. 4e, f). To further demonstrate that Nrf2/ARE signaling pathway was involved in KAT6A-mediated improvement of stemness of OBMSCs, KAT6A was also overexpressed in OBMSCs in the content of Nrf2 siRNA, and ROS level was increased after Nrf2 inhibition in KAT6A-overexpressed OBMSCs (Fig. 4g). The proliferative capacity and the colony-forming ability were also inhibited after Nrf2 inhibition (Fig. 4h, i). Meanwhile, qRT-PCR and alizarin red staining analysis showed that KAT6A regulated osteogenic differentiation of aging BMSCs through Nrf2/ARE signaling pathway (Fig. 4j, k).

### KAT6A regulated osteogenesis of aging BMSCs through Nrf2/ARE signaling pathway in vivo

**Fig. 5:**
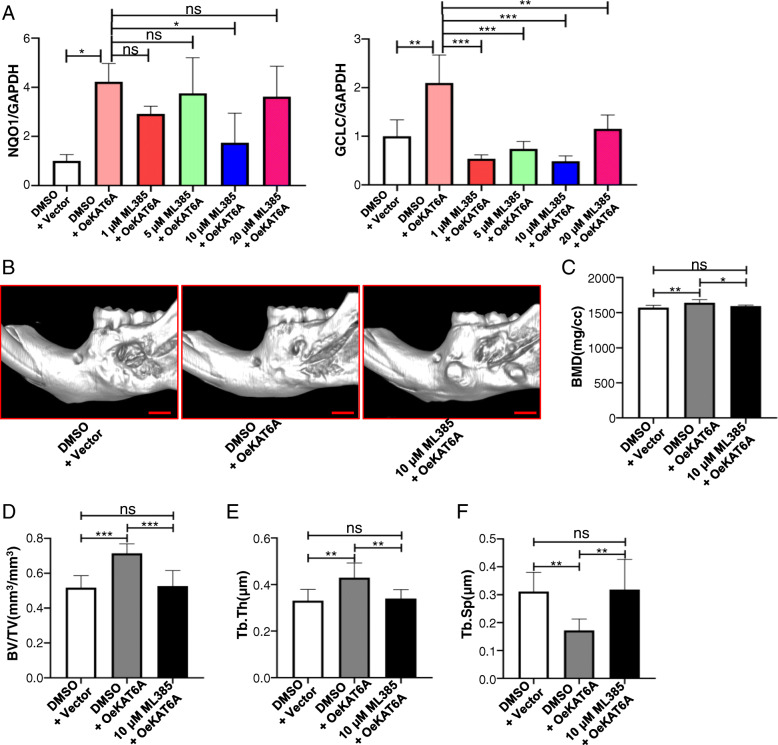
KAT6A regulated osteogenesis of OBMSCs through Nrf2/ARE signaling pathway. **a** OBMSCs were treated with “DMSO + vector” or “DMSO + KAT6A plasmid” or KAT6A plasmid with 1 μM, 5 μM, 10 μM, or 20 μM ML 385 in sheet-induction medium for 7 days, then qRT-PCR was performed to explore the expressions of *GCLC* and *NQO1* (*n* = 3)*.* **b** OBMSCs were treated with “DMSO + vector” or “DMSO + KAT6A plasmid” or KAT6A plasmid with 10 μM. ML 385 in sheet-induction medium for 14 days, then the sheets were filled in the defects. After 6 weeks, micro-CT was performed to observe the defects (*n* = 7 to 8). **c**–**f** OBMSCs were treated with “DMSO + vector” or “DMSO + KAT6A plasmid” or KAT6A plasmid with 10 μM. ML 385 in sheet-induction medium for 14 days, then the sheets were filled in the defects. After 6 weeks, micro-CT was performed to observe the indexes of bone mineral density (BMD), bone volume/total volume (BV/TV), trabecular thickness (Tb.Th), and trabecular separation (Tb.Sp). For each index, the exact value of *n* in the group of “DMSO + vector” or “DMSO + KAT6A plasmid” or KAT6A plasmid with 10 μM ML 385 was 8, 7 and 6 for BMD, and 8, 7 and 7 for BV/TV, Tb.Th and Tb.Sp. Scale bar 2 mm

In vivo study was also performed to demonstrate the osteogenesis of BMSCs after KAT6A and/or Nrf2 modification. ML 385, a specific Nrf2 inhibitor, was used to inhibit Nrf2/ARE signaling pathway, and the concentration of ML 385 was chosen according to previous researches [24, 25]. Then, OBMSCs were treated with “DMSO + vector” or “DMSO + KAT6A plasmid” or KAT6A plasmid with 1 μM, 5 μM, 10 μM, or 20 μM ML 385 in a sheet-induction medium for 7 days, and qRT-PCR analysis showed that 10 μM ML 385 could significantly inhibit Nrf2/ARE signaling pathway of KAT6A-overexpressed OBMSCs (Fig. 5a). Thus, 10 μM ML 385 was used in our study. After sheet formation, sheets were filled in the alveolar bone defects. After 6 weeks, micro-CT was performed to observe the indexes of bone mineral density (BMD), bone volume/total volume (BV/TV), trabecular thickness (Tb.Th), and trabecular separation (Tb.Sp). The results showed that BMD, BV/TV, Tb.Th, and Tb.Sp were all improved in KAT6A-overexpressed OBMSC groups compared to those in the vector-overexpressed OBMSC group (Fig. 5b–f). However, compared to KAT6A-overexpressed OBMSCs, these indexes in KAT6A-overexpressed followed by ML385 treatment OBMSC group were decreased, suggesting that KAT6A promoted osteogenesis of OBMSCs through Nrf2/ARE signaling pathway.

## Discussion

**Fig. 6:**
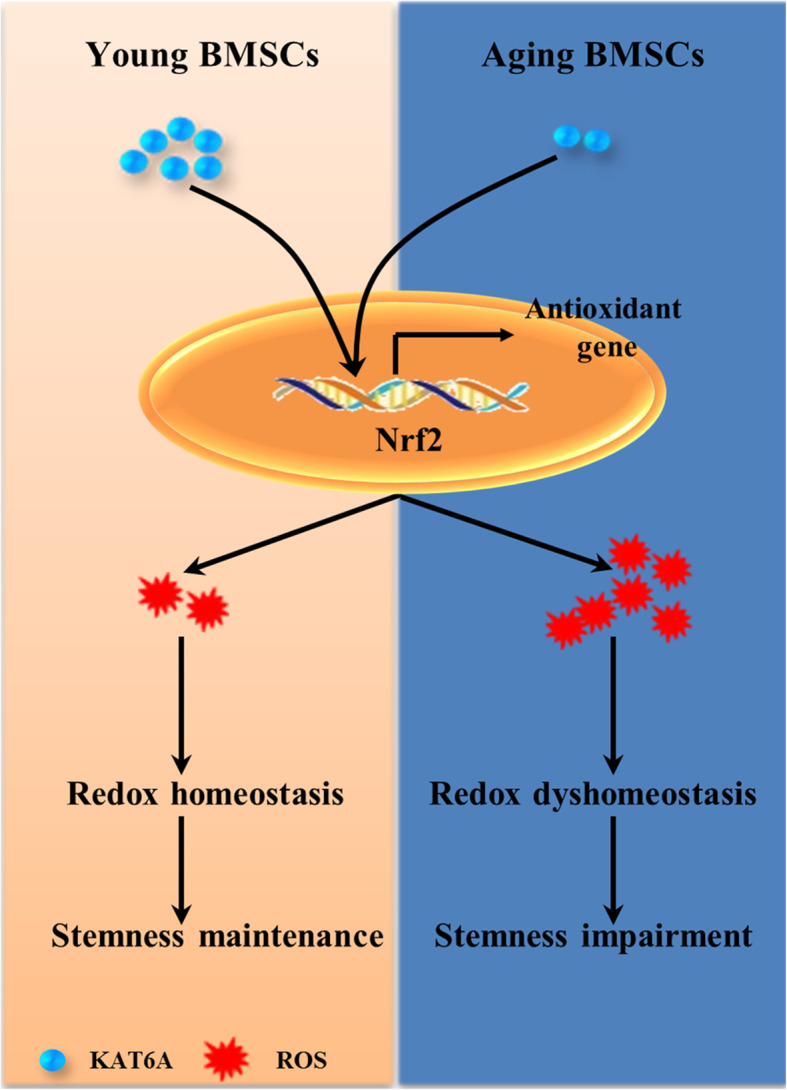
Graphic summary shows the mechanism of decreased KAT6A-mediated stemness impairment in aging BMSCs. KAT6A was downregulated in aging BMSCs; this downregulation resulted in Nrf2/ARE signaling pathway inhibition and ROS accumulation, which further led to stemness impairment

In our study, the relationship between KAT6A and Nrf2/ARE signaling pathway in the aging process of BMSCs was uncovered, which has not been recognized previously. As summarized in Fig. 6, our study firstly found that KAT6A was downregulated in the aging process of BMSCs, and demonstrated that KAT6A downregulation in OBMSCs could result in Nrf2/ARE signaling pathway inhibition and ROS accumulation, which further led to stemness impairment.

Osteoporosis is one important manifestation of aging, and many studies have shed light on the decreased stemness of BMSCs and proved that multifarious mechanisms including signaling pathways including NAD^+^–Sirt1 [26] and Notch [27], miRNA [28, 29], and organelle dysfunction [30] contributed to the decline during cellular senescence. Epigenetic alteration is one primary hallmark of aging, and the progressive accumulation of epigenetic alteration could lead to stem cell senescence through changing genomic instability and gene expression profiles. Histone acetylation, which is determined by HATs and histone deacetylases (HDACs), is an important part of aging-related epigenetic alteration, and numerous studies have shown that histone acetylation modification could affect stem cell senescence. Some studies demonstrated that HDAC inhibitor (HDACi) could improve aging-related phenotype by increasing histone acetylation level [31–33], while others found that HDACi could induce cellular senescence [34, 35]. The nonspecificity of the drugs and the specificity of different HATs and HDACs may partly account for this. Therefore, locating the key histone acetylation-related enzymes in the aging process is of great significance. In our study, decreased acetylation levels of lysine and downregulated histone H3 acetylation in OBMSCs were found, which suggested that histone H3 acetylation may play an important role in the decreased stemness of OBMSCs. The KAT6A gene, which was firstly discovered in t(8;16)(p11;p13) chromosome translocations, can specifically acetylate histone H3. KAT6A has been widely known for its contribution in leukemogenesis due to its histone acetyltransferase activity [36]. And beyond that, KAT6A could also regulate Runx2, an osteogenic transcriptional factor [37], and promote the osteogenic differentiation of periodontal ligament stem cell [22]. Recent research showed that inhibition of KAT6A activity could induce the senescence of mouse embryo fibroblasts through p16INK4A–p19ARF pathway [10], indicating the potential effect of KAT6A on cellular senescence. In our study, KAT6A was found to be downregulated. Through gain- and loss-of-function experiments, decreased KAT6A expression was proven to have resulted in the decreased stemness of OBMSCs.

ROS generated from normal metabolism or external stimulus can accumulate during the aging process. They cause damage to DNA, protein, and organelle [15] and perturb cell proliferation [38], induce apoptosis [39], and impair differentiation [40]. Nrf2/ARE signaling pathway is responsible for oxygen radical scavenging, and Nrf2 deficiency can disrupt bone metabolism as Nrf2−/− mice exhibited increased bone resorption and reduced bone formation [41]. Numerous studies have confirmed disruption of Nrf2/ARE signaling pathway during the aging process [15, 42]. One study reported that cellular localization of Nrf2 was changed in replicative senescence, and increasing nuclear Nrf2 could improve stemness of late-passage BMSCs [43]. Another study found that impaired activity of Nrf2/ARE signaling pathway was a driver mechanism in Hutchinson-Gilford progeria syndrome through high-throughput RNAi screening, and Oltipraz, a Nrf2-activating agents, could ameliorate the progeroid phenotype [42]. The connection between HATs and Nrf2/ARE signaling pathway has been reported: One study showed that coactivator P300 can regulate sodium butyrate-mediated transcriptional activation of Nrf2 by binding to the Nrf2 gene promoter region [44]; another study found that histone acetyltransferase MOF could increase nuclear retention of Nrf2 in 293 T cells and improve antioxidative responses [21]. Given these, KAT6A was speculated to regulate stemness through Nrf2/ARE signaling pathway. Firstly, KAT6A was demonstrated to regulate Nrf2/ARE signaling pathway. Further study showed that inhibited Nrf2/ARE signaling pathway contributed to the decreased stemness of OBMSCs and KAT6A could improve stemness through the Nrf2/ARE signaling pathway. What is more, in vivo study was also performed to investigate whether KAT6A-mediated Nrf2/ARE signaling pathway could regulate bone formation of OBMSCs, an index reflecting the stemness of OBMSCs. Results showed that upregulated KAT6A in OBMSCs could promote the osteogenesis ability of OBMSCs, and inhibition of Nrf2/ARE signaling pathway could reverse KAT6A-mediated improvement of osteogenesis ability of OBMSCs.

## Conclusions

This study confirmed that aging BMSCs lost stemness compared to YBMSCs and demonstrated KAT6A-mediated decreased stemness of aging BMSCs and clarified the mechanism of KAT6A by regulating the Nrf2/ARE signaling pathway. With respect to research significance, this study not only finds a new mechanism for the decreased stemness of aging BMSCs but also provides a feasible strategy for the treatment of age-related osteoporosis.

## Supplementary Information

**Additional file 1: Table S1.** Sequence of primers for qRT-PCR.

**Additional file 2: Figure S1.** The stemness of OBMSCs was decreased compared to YBMSCs. (a) CCK-8 was performed to explore the proliferative capacity of YBMSCs and OBMSCs (*n* = 3). (b) The colony-forming abilities of YBMSCs and OBMSCs were explored by crystal violet after culture for 12 days (n = 3). (c) After osteogenic induction for 14 days, expressions of osteogenic-related genes of Funx2, OCN and BMP2 were detected in YBMSCs and OB < SCs (n = 3). (d) After osteogenic induction for 21 days, alizarin red staining was performed in YBMSCs and OBMSCs (n = 3). *P < 0.05; **P < 0.01; ***P < 0.001.

## Data Availability

The datasets used and/or analyzed during the current study are available from the corresponding author on reasonable request.

## Abbreviations

BMSCs: Bone marrow-derived mesenchymal stem cells
YBMSCs: BMSCs from the young
OBMSCs: BMSCs from the old
HATs: Histone acetyltransferases
Runx2: RUNX family transcription factor 2
ROS: Reactive oxygen species
Nrf2: Nuclear factor erythroid 2-related factor 2
CFU-F: Colony-forming unit-fibroblastic
CCK-8: Cell Counting Kit-8
qRT-PCR: Quantitative reverse transcription polymerase chain reaction
OCN: Osteocalcin
BMP2: Bone morphogenetic protein 2
GAPDH: Glyceraldehyde-3-phosphate dehydrogenase
HDACs: Histone deacetylases
HDACi: HDAC inhibitor
