# Supplementary material for: KAT6A regulates stemness of aging bone marrow-derived mesenchymal stem cells through Nrf2/ARE signaling pathway
Source: Stem Cell Res Ther. 2021 Feb 4;12:104. doi: 10.1186/s13287-021-02164-5 (PMC7860225; doi:10.1186/s13287-021-02164-5)
Supplement: Supplementary file 1 — Additional file 1: Table S1. Sequence of primers for qRT-PCR. [file 13287_2021_2164_MOESM1_ESM.docx]

| mRNA/primer | Forward (5’-3’) | Reverse (5’-3’) |
| --- | --- | --- |
| GAPDH | GCACCGTCAAGGCTGAGAAC | TGGTGAAGACGCCAGTGGA |
| Runx2 | CGGAATGCCTCTGCTGTTATG | AAGGTGAAACTCTTGCCTCGTC |
| OCN | GCCACCGAGACACCATGAGA | AGGCTGCACCTTTGCTGGAC |
| BMP2 | ACCCGCTGTCTTCTAGCGT | TTTCAGGCCGAACATGCTGAG |
| Nrf2 | TCAGCGACGGAAAGAGTATGA | CCACTGGTTTCTGACTGGATGT |
| NQO1 | GAAGAGCACTGATCGTACTGGC | GGATACTGAAAGTTCGCAGGG |
| GCLC | GGAGGAAACCAAGCGCCAT | CTTGACGGCGTGGTAGATGT |
| KAT6A | TTCACCAGCAGTTACGATTG | CAGAAACTACAGATGGGGATT |

GAPDH: glyceraldehyde-3-phosphate dehydrogenase; RUNX2: RUNX family transcription factor 2; OCN: osteocalcin; BMP2: bone morphogenetic protein 2; Nrf2: nuclear factor, erythroid 2 like 2; NQO1: NAD(P)H quinone dehydrogenase 1; GCLC: glutamate-cysteine ligase catalytic subunit
