# Supplementary figures and images for: KAT6A regulates stemness of aging bone marrow-derived mesenchymal stem cells through Nrf2/ARE signaling pathway
Source: Stem Cell Res Ther. 2021 Feb 4;12:104. doi: 10.1186/s13287-021-02164-5 (PMC7860225; doi:10.1186/s13287-021-02164-5)

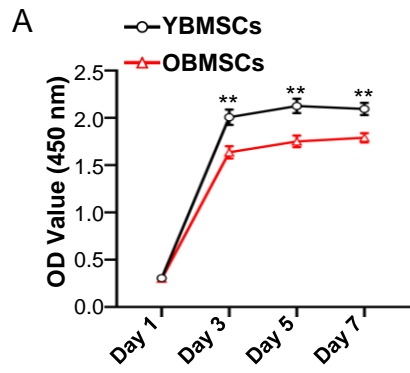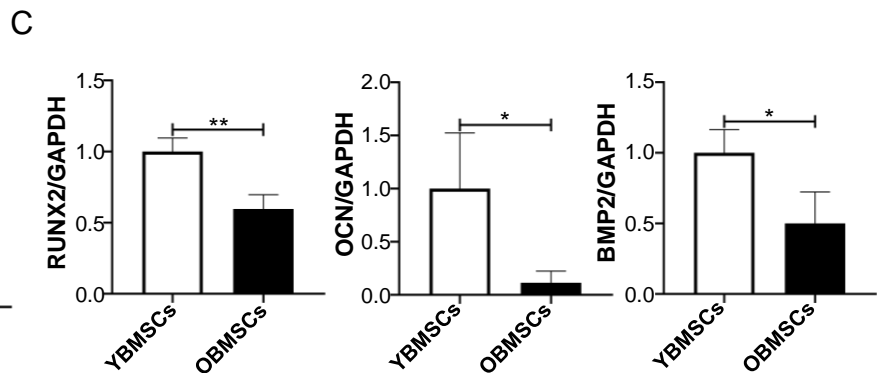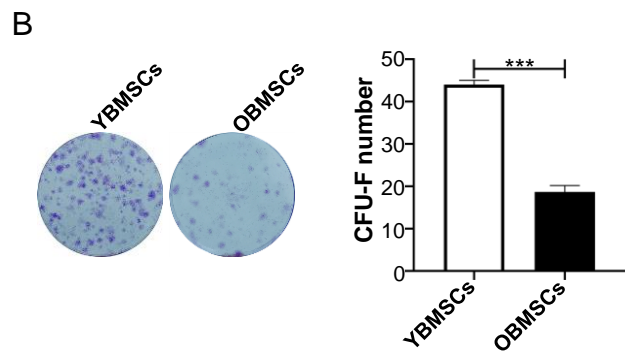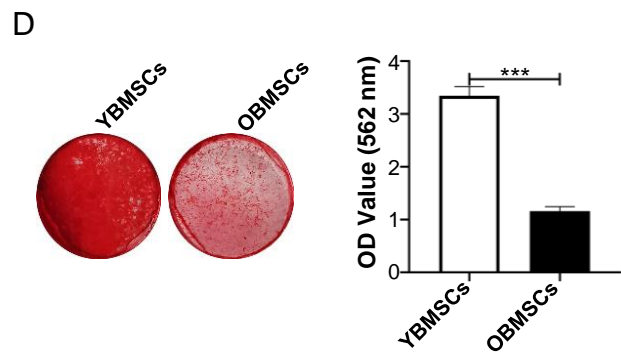

Supplement: Supplementary file 2 — Additional file 2: Figure S1. The stemness of OBMSCs was decreased compared to YBMSCs. (a) CCK-8 was performed to explore the proliferative capacity of YBMSCs and OBMSCs (n = 3). (b) The colony-forming abilities of YBMSCs and OBMSCs were explored by crystal violet after culture for 12 days (n = 3). (c) After osteogenic induction for 14 days, expressions of osteogenic-related genes of Funx2, OCN and BMP2 were detected in YBMSCs and OB < SCs (n = 3). (d) After osteogenic induction for 21 days, alizarin red staining was performed in YBMSCs and OBMSCs (n = 3). *P < 0.05; **P < 0.01; ***P < 0.001. [file 13287_2021_2164_MOESM2_ESM.pdf]
